# Mobility and postural limitations perceived by transtibial amputees undertaking agricultural activities: a qualitative study

**DOI:** 10.1080/07853890.2023.2258915

**Published:** 2023-09-22

**Authors:** Y. Ortega Bedoya, V. Mejía Londoño, E. Rendón Vélez, F. Valencia Legarda, J. A. Plata-Contreras

**Affiliations:** a School of Applied Sciences and Engineering, Universidad EAFIT, Medellín, Colombia; b Department of Physiotherapy, María Cano University Foundation, Medellín, Colombia; c School of Medicine, University of Antioquia, Medellín, Colombia

**Keywords:** Transtibial amputation, prosthesis, farmer, mobility problems, postural limitations, agricultural activities, content analysis

## Abstract

**Purpose:**

In Colombia, 98% of landmines occur in rural areas, where the main victims of amputation are farmers. The challenges these amputees face in their agricultural work remain unknown. The aim of this study is to determine the mobility and postural limitations these farmers face in carrying out their daily activities.

**Method:**

Forty-nine participants meeting the following criteria were interviewed: transtibial amputee, 18 years and over, performs agricultural labour and wears the prosthesis daily. Subsequently, the interview transcripts were subjected to a content conventional analysis and responses were organized according to the abstraction process to identify categories and subcategories of the problems.

**Results:**

Main problems reported were walking on sloping, uneven and wet terrain, problems associated with the stump skin, squatting, kneeling, using vehicles or animals for transportation and carrying objects over 30 kg. Postures such as sitting, running, jumping, and standing on tiptoes were mentioned less frequently.

**Conclusions:**

In conclusion, the prostheses worn by transtibial amputee farmers are not suitable for working on sloping and uneven terrain, nor for performing postures such as kneeling or squatting. These postures are very common in agricultural and livestock tasks in countries with mountainous areas such as Latin American countries. The recognition of problems reported by farmers transtibial amputees, may help to improve the design of prostheses so that they meet the needs of this population and decrease secondary injuries associated with prosthetic use. This information is useful to identify compensatory postures that facilitate prosthetic adaptation and rehabilitation for amputees.

## 1. Introduction

Disability is one of the biggest problems in the world; It is estimated that more than one billion people experience some kind of disability. This corresponds to about 15% of the world population [1,2]. According to WHO reports, between 0.25% and 1.25% of the world’s population has physical disabilities associated with amputation or loss of a body part [3]. About 159,000 amputations are performed annually [4], among the main causes are vascular problems related to diabetes, traffic accidents, infections, and cancer. Traumatic injuries account for about 45% of all amputations [5].

The Colombian Association of Physical Medicine and Rehabilitation estimates that the incidence of amputation in the country is 200 to 300 people per 100 thousand inhabitants [6]. Colombia is the country with the second highest incidence of injuries caused by anti-personnel landmine (APL) and unexploded ordnance [4]. By September 2022, according to the Antipersonnel Mines Observatory, there have been 12,264 victims of APL and unexploded ordnance, of which 81% (9,910) were injured, the rest dead, mainly men [7]. An estimated 70% of APL and unexploded ordnance injuries result in limb amputation [4]. This consequence of the armed conflict is added to the other main causes of amputation in our country.

Rural amputees are a population that needs attention. The main civilian victims of APL are farmers since 98% of explosions occur in rural areas [8]. In addition, health centres are far from rural areas, so diseases such as diabetes mellitus or peripheral vascular disease can aggravate in this population due to lack of opportune clinical services [9]. The most common amputations are lower limb amputations, i.e. amputations of limbs below the hip, specifically transtibial amputations (below-knee amputations) [10]. Similarly, in developed countries such Germany, where there are no explosive devices such as APL, most amputations occur due to neurovascular diseases caused by factors such as diabetes, where most of these amputations occur in men and at the level of foot and toe [11]

Furthermore, farming injuries are two and a half times more likely to end in amputation than any other type of injury. According to the National Agricultural Safety Database, to lose a limb is a risk farmers face every day [12]. People who live in rural areas and work in farming and ranching, usually in remote locations far from the cities have unique needs that are not well served by current prosthetic devices. In addition, these farmers and ranchers who wear prostheses are at risk for secondary injuries from falls, entanglement of the prosthesis with farm machinery, injury from overuse of the sound limb, and residual limb injuries [13,14].

Most poor people live in rural areas in developing countries and depend on agriculture for their livelihood, approximately 85% of rural dwellers depend on agriculture [15]. Having access to an adequate prosthesis when undertaking agricultural activities is difficult because they are generally manufactured with materials and technologies that can be expensive and that make these prostheses unaffordable for low-income amputees. In addition, the adaptation of the prosthesis is a long process that is not evident in a few days, but in months and many of the existing prostheses are not adapted to the activities of a farmer. In some of the investigations carried out in countries similar to Colombia, rural users reported that durability, inadequate material tenacity and low resistance to environmental factors (i.e. resistance to water and mud) are important problems that have not been solved in current prosthetic devices [16]. Durability problems have been reported for components such as the foot prosthesis and the gradual deterioration of the prosthesis in general due to the harsh conditions in rural areas [17,18]. This continuous deterioration and poor fit of the prosthesis cause people to stop using the prosthetic device.

There are few studies reported in the literature on the problems that amputees in the agricultural sector have in the performance of their daily activities. Among the most relevant publications is that of Waldera et al. who conducted an interview-based study in the United States where they found the needs of farmers and ranchers with amputations. Although their sample of interviewees was considerable (40 amputees), only 15 had transtibial amputation, which made it impossible to categorize the needs and problems mentioned by the interviewees and evidenced the need for a larger sample of subjects to adequately capture this diversity. [14]. Currently, there are no similar studies for Latin American countries or low-income countries. Andrysek, in his review about lower limb technologies in the developing world, reported on the low number of publications made by these countries in peer-reviewed journals. In addition, on many occasions, the publications do not cover the testing of aspects such as evaluation of patient, results in terms of durability, maintenance, and cultural environment [17]. Sayeed et al. studied factors influencing access to and participation in rehabilitation for people with lower limb amputation (LLA) in developing countries in East, South and Southeast Asia. They found that improving rehabilitation services for this context requires supportive environments, accessible transport, social and economic security, and increased awareness, backed by appropriate policy [19]. Therefore, the aim of this study is to analyse and categorize the mobility and postural limitations of farmers and ranchers with transtibial amputations when performing their work, from the users’ perspective.

## 2. Materials and methods

### 2.1. Participants

This study was approved by the ethics committee of the Fundación Universitaria María Cano (extraordinary session #1 of 2020). In the first instance, we searched for people with transtibial amputation in the databases of institutions that manufacture and adapt lower limb prostheses in the city of Medellín and its metropolitan area (Mahavir Kmina Corporation, Orthopraxis and TAO). Telephone calls were made to the patients, and a survey was carried out (Annex 1), verifying that they met the following inclusion criteria: transtibial amputee, 18 years of age and over, who performs agricultural work and uses his prosthesis most of the day. Those respondents who met the inclusion criteria were invited to participate in the study.

### 2.2. Data collection

A semi-structured interview was conducted to inquire about the mobility and postural limitations of transtibial amputees while doing agricultural work, as well as the functional problems of prostheses in this context. In this study we will only present data on the mobility and postural limitations. Before conducting the interview, all participants gave their informed consent verbally^1^ or in writing, where they authorized to record the interviews, as well as to use the information provided for this research.

The interviews were conducted by the first author (YO) between March 2020 and January 2022, in person, at the institutions where the prostheses are manufactured, or telephonically. Each interview lasted 45 to 60 min.

The semi-structured interview instrument has 5 main sections (see Annex 2): the first section is introductory, where the purpose of the call is explained to the patient and consent is requested to record the conversation and use the information provided for research purposes. In the second section, questions are asked about the demographic and contact information of the interviewees, followed by a section inquiring about relevant amputation information such as level, side, cause and date. The fourth section asks about the patient’s experience with prosthesis use and the mechanical status of their current prosthesis (type, age, materials, state of repair). In the last section of the interview, we inquired about the agricultural activities that the amputee performs daily, asking for clarity about the use, adaptation and feeling of comfort of the prosthesis during the execution of daily agricultural work. The audios of each interview were digitally saved and transcribed verbatim in their entirety (by YO and VM)^2^. The interviews were conducted and analysed in Spanish, as it is the native language of all the participants in this study (Colombians).

### 2.3. Data analysis

This study was conducted using conventional or inductive content analysis, whose objective is to describe a phenomenon, mainly when the existing literature on the subject is limited [20]. With this method, an open coding of the information was performed, and categories emerged from the data, going from the specific to the general, so that particular instances are observed and then combined into a broader whole or general statement. Based on the recommendations of Elo and Kingüas [21], the following methodology was developed.

### 2.4. Preparation phase

The literal transcription of each of the semi-structured interviews applied was selected as the unit of analysis, analysing only the manifest content. All the audios and transcripts were entered into the NVIVO software, and all the content analysis was carried out there. Each of the authors listened to and read at least 20% (*n* = 8) of the audios and interview transcripts on repeated occasions, to familiarize themselves with the data and make initial annotations.

### 2.5. Organizational phase

Two of the authors (VM and YO) obtained codes in an open-ended manner by initially highlighting exact phrases from the text that capture key thoughts from transtibial amputees about discomfort associated with mobility and postural limitations when performing agricultural work. Coding sheets were then created and discussed in several meetings by all authors until a consensus was reached, each giving their assessment from their area of expertise: Bioengineering (YO), Product Design Engineering (VM), Physical Medicine and Rehabilitation (JP), Physiotherapy (FV), and Mechanical Engineering (ER). The initial labels were grouped under higher order headings. The refinement of the categorization was performed by the first two authors, consulting with the other investigators whenever necessary. This recategorization process was repeated until a universal classification was obtained for the problems associated with the use of prostheses when undertaking frequently performed activities. The variety in the professional experience of the co-researchers helped to minimize the risk of bias, and discussions were held to avoid subjective thinking. The researchers ensured that the analysis was data driven. All content analysis was performed with the information in the native language, Spanish. However, for the purpose of reporting the results in this article, the analysis abstraction and participant quotes were translated as reliably as possible.

## 3. Results

### 3.1. Attributes and characteristics of people interviewed

Table 1 shows the main characteristics of the 49 interviewees who met the inclusion criteria. 8.2% of the interviewees were women (*n* = 4), 4.1% suffered an amputation during the year of the interview (*n* = 2), 12.2% have been using prosthesis for less than one year (*n* = 6), 6.1% (*n* = 3) of the interviewees are older adults (over 60 years old), most of the patients alternate their daily activities between livestock and agriculture (*n* = 23 (46.9%)).

**Table 1.** Interviewee profile.

Attribute		Quantity	Percentage (%)
Age	18 to 26 years old	4	8,2
	27 to 36 years old	13	26,5
	37 to 46 years old	14	28,6
	47 to 59 years old	15	30,6
	Older than 60 years	3	6,1
Gender	Male	45	91,8
	Female	4	8,2
Amputation time	Less than 1 year	2	4,1
	Between 1 and 5 years	9	18,4
	Between 6 and 10 years	11	22,4
	Between 11 and 15 years	9	18,4
	More than 15 years	18	36,7
Time wearing prostheses	Less than 1 year	6	12,2
	Between 1 and 5 years	8	16,3
	Between 6 and 10 years	9	18,4
	Between 11 and 15 years	11	22,4
	More than 15 years	15	30,6
Amputation cause	Anti-personnel landmine (APL)	8	16,3
	Farm accident	16	32,6
	Traffic accident	9	18,4
	Neurovascular and other diseases	16	32,6
Agricultural and livestock activities	Agriculture	16	32,6
	Livestock	7	14,3
	Agriculture and livestock	23	46,9
	Other*	3	6,1

* People who occasionally worked in agriculture and livestock farming but not as a main activity.

### 3.2. Data analysis

**Figure 1.**
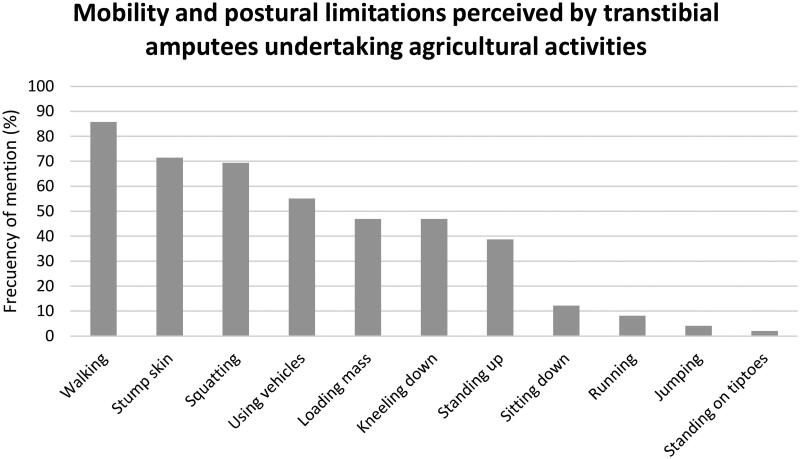
Frequency of mobility and postural limitations perceived by transtibial amputees undertaking agricultural activities.

Figure 1 shows the number of amputees who reported problems associated with static and dynamic postures performed during their workday in the field. The following sections explain in detail the agricultural activities and their most critical tasks associated with the problems found after the abstraction process. Some participant quotes illustrating the categories are presented. They are identified by the ID of the interviewed participant (S) and his or her age.

#### 3.2.1. Walking on different types of terrain

Forty-two (85.7%) of the respondents, indicated that they had physical problems associated with walking on different types of terrain, including walking on sloping, uneven, wet and swampy surfaces, as well as in tall grass. Transtibial prosthesis users reported risks of falling or loss of balance while harvesting, spraying, cleaning coffee, planting corn, or weeding. Harvesting coffee is difficult due to the topography of some coffee growing areas, which are characterized by being planted on sloping terrain.

“When harvesting, it depends on the terrain. If the terrain is a bit steep, it makes it difficult for me to hold myself up. There are parts where I must kneel. If the terrain is flat, I work normally. But if it’s very steep, I don’t like it, because I get tangled up a lot and I feel that Ím going to fall.” (S46, age29)

On the other hand, rice planting requires the terrain to be flooded either rain-fed or irrigated, so farmers who are engaged in this work report having problems with the adjustment of the prosthesis and risk of falling when having to walk on this type of terrain.

“Because it (the rice field) is always full of water or mud. And if I must stay there all day long in the water and mud, the prosthesis does not last long because the screws rust. Everything gets muddy and when you walk you feel heavy as if the prosthesis had a chewing gum sticking it to the ground. You feel that you are going to fall”. (S30, age38)

Another activity that is difficult on sloping, uneven terrain and with tall grass is spraying, since amputees must carry a pump with liquid weighing approximately 25 kg on their backs. While pumping with one hand, they spread the liquid over the crops with the other. It is demanding for amputees to maintain balance on this terrain when spraying.

“… on hills and slopes… so that’s where it’s bad for me because I don’t have anything to lean on. I have nothing to lean on and the weight is constant on my back (the weight of the pump), so I don’t last long. I usually work about half the time”. (S1, age28)

“… it is difficult to walk on hills and slopes. with the pump you can’t do it. For example, in my case, sometimes I can slip and fall far”. (S32, age46)

Finally, they indicated that they have problems with weeding on uneven surfaces.

“… when weeding in irregular terrains, there are moments when I lose my balance, because I feel that the prosthesis weighs a lot. It is very heavy, and that is when I lose my balance or stumble”. (S10, age56)

**Figure 2.**
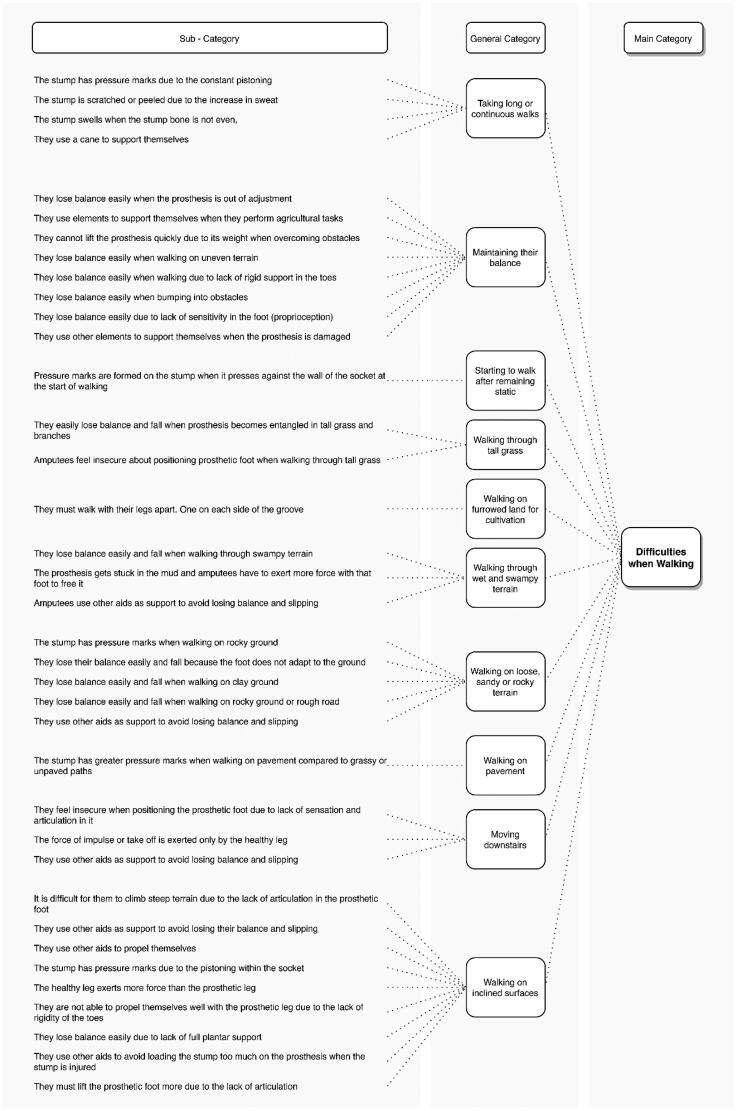
Problems reported by transtibial amputees when walking on different terrains.

Figure 2 shows the abstraction of the problems reported by amputees when walking during their workday.

#### 3.2.2. Problems in the stump skin

**Figure 3.**
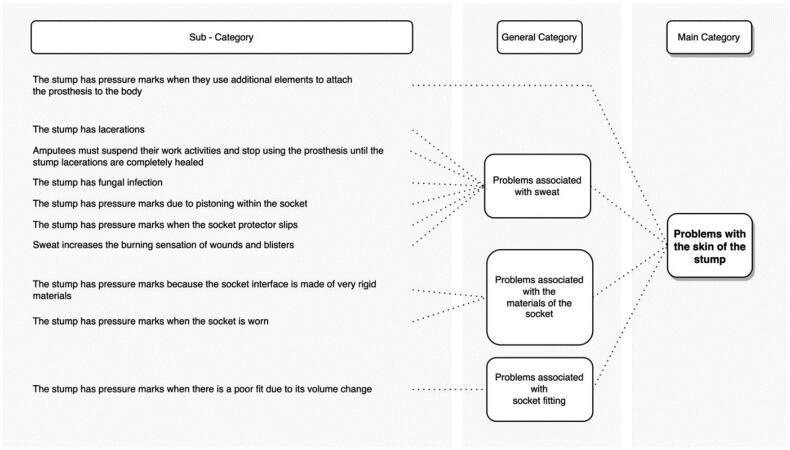
Problems that transtibial amputees present on the stump skin.

The skin of the residual limb was the second most frequently mentioned category by the interviewees, 71.4% of the amputees who reported skin discomfort due to the postures of agricultural work. Figure 3 shows the categories and subcategories found after the abstraction process. Among the main problems were lacerations, pressure marks, blisters, and fungus infections due to sweating, wear or tear of the socket materials or changes in the volume of the stump. Amputees perceived these problems while performing agricultural activities such as weeding and fertilizing coffee.

“Well, yes, let’s say I’m weeding in a hurry, and I get very agitated, I sweat. If I’m fertilizing and I fertilize fast, I sweat. I mean, the sweating problem is too high. I am very prone to what I told you, abscesses, and ulcers on my residual limb”. (S1, age28)

15 respondents mentioned that they must suspend their activities and stop using the prosthesis until the skin of the stump heals completely. A situation that harms their work productivity and therefore their economy.

“… just on the residual limb, because suddenly a pore is blocked and it becomes like an abscess, so you have to stop wearing the prosthesis for about fifteen days while it is drained, while it heals, while I take some medication”. (S14, age40)

“When it blisters a lot (the residual limb) then I take it off (prosthesis) and I don’t work. I take a rest for 1 or 2 days and then I rub Vaseline all the time and it heals immediately”. (S25, age40)

#### 3.2.3. Squatting

**Figure 4.**
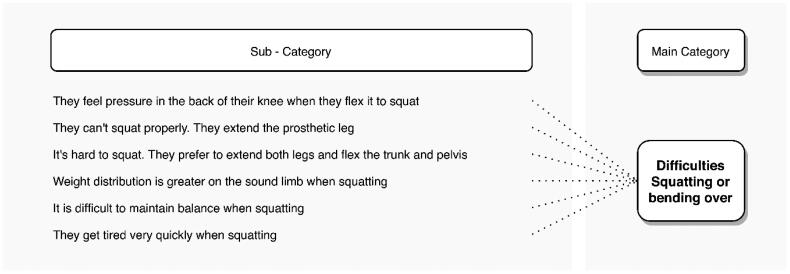
Problems reported by transtibial amputees while squatting.

**Figure 5.**
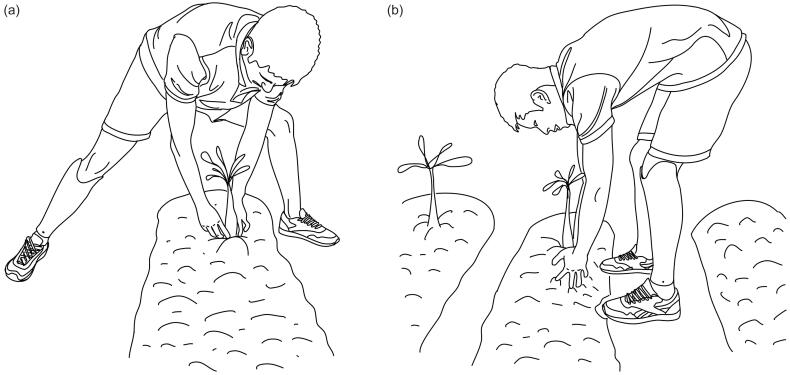
Postures adopted by transtibial amputees to reach for objects on the floor.

The static posture most indicated as problematic by the interviewees is the squatting posture, with 69.4%. Figure 4 shows the subcategories obtained from the interview excerpts analysed. Transtibial amputees avoid this posture and instead extend the knee of the amputated limb, abduct the hip and flex only the knee of the sound limb, as illustrated in Figure 5a. Others opt to leave both knees extended, abduct the hips, and flex the trunk more than 60° to reach the floor (Figure 5b), generating problems in the thoracic and lumbar areas. For their part, amputees who manage to squat down report that the back of the knee is pressed by the upper part of the socket, and they also find it difficult to maintain their balance in this position, so they tire quickly. In general, they rely more on the sound leg when they manage to squat. This position is very common in agricultural activities that require interaction with the ground, such as weeding, planting sugar cane, beans, coffee, potatoes, and harvesting low crops such as beans and some fruits.

“The difference is that when planting corn, I don’t have to bend down and when growing coffee, I do. When I am planting coffee, I must bend down a lot to place the plant, the seed, the seedling, the bush, the hole and …. and cover it, plant it… I bend my spine”. (S10, age56)

“Yes, planting bean is more uncomfortable because this (the knee) doesn’t allow you to bend down. It doesn’t work, because this down here (the socket) is straight, so it doesn’t work. Of course, farmers plant beans crouching down, but I plant them standing up because of my prosthetic leg. I bend down a little bit and then I plant”. (S24, age43)

“I always have to bend down, because the furrow is on the ground and I have to bend to sow, to harvest, for all activity …I bend only at the waist, I bend, and I work bent over”. (S11, age54)

#### 3.2.4. Use of vehicles and animals as a means of transport

**Figure 6.**
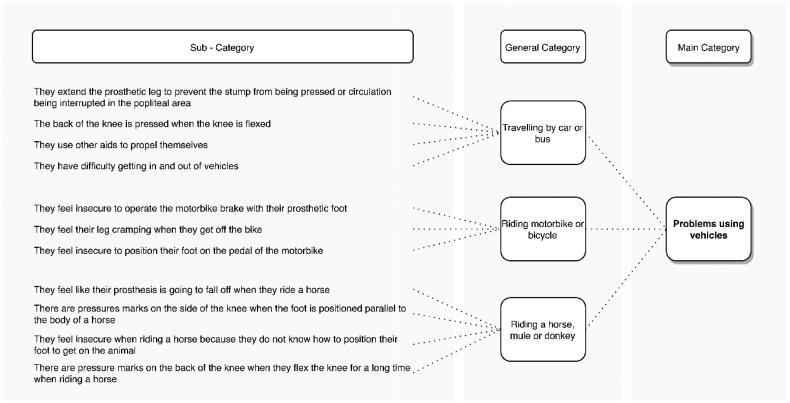
Problems reported by transtibial amputees when using vehicles and animals as a means of transport.

**Figure 7.**
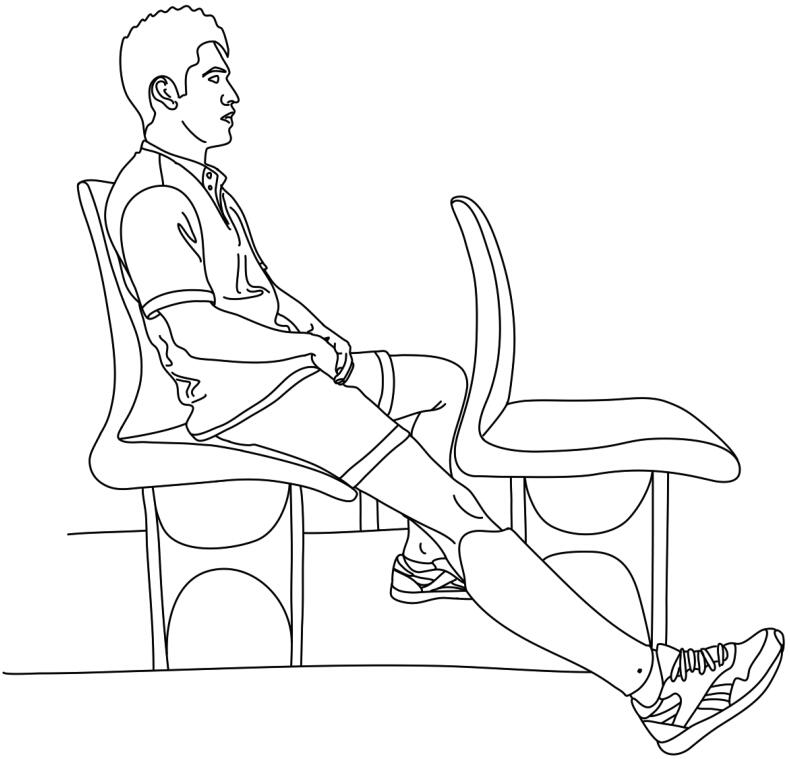
Posture adopted by transtibial amputees when traveling on buses.

This category grouped the problems mentioned by the amputees when using their means of transportation, whether by car, motorbike, or horse. A total of 55.1% of the amputees reported problems when moving around in vehicles. Figure 6 shows the categories and subcategories of problems associated with the use of means of transport in agricultural tasks. The most reported is associated with traveling in vehicles with low seats, such as automobiles, during long trips. They indicated that they must keep the knee of the amputated leg extended to prevent the stump from being pressed by the socket or interrupting blood circulation in the popliteal area (Figure 7). In this case, some prefer to remove the prosthesis during the journey, while others choose to keep it on all the time to facilitate movement in case of emergency.

“On a long trip it is very bad in a car because I must go with my leg straight. If my leg is flexed, I get very tired. For example, if I’m in a private car in the back of the car it’s very bad for me. On the other hand, if I am in the front seat, and my leg is stretched out like this, I can go as far as I want”. (S41, age48)

“In the bus I must sit on the side near the corridor to be able to stretch my leg; on the left side to be able to keep the prosthesis stretched in the corridor”. (Left leg amputee) (S44, age39)

Some of the amputees mentioned that they could ride a motorbike. When using this means of transportation, they show problems such as insecurity to operate the motorbike brake and to place the prosthetic foot on the motorbike pedal due to the lack of proprioception in this area.

“Not on a motorbike. It scares me. I don’t feel the pedal. When I ride a motorbike, I don’t feel where I place my foot. I don’t feel the prosthesis”. (S37, age54)

When they are traveling on horseback, they feel that the prosthesis is going to come loose. The lateral part of the knee presents pressure marks when the prosthetic leg is parallel to the horse’s body, after riding.

“It’s more complicated when I ride a motorbike or a beast because that’s where it tries to come off (the prosthesis)”. (S16, age28)

These situations are common to several agricultural activities, as they need to use these means of transport to move to their work area, to transport themselves in the field, to move food or crops and to herd livestock.

#### 3.2.5. Loading mass above 30 kg

**Figure 8.**
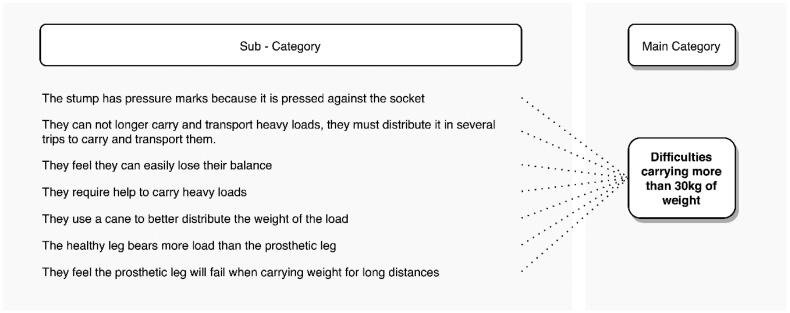
Problems reported by transtibial amputees when carrying a mass above 30 kg.

Another problem mentioned by transtibial amputees occurs when attempting to carry masses greater than 30 kg. Figure 8 shows the abstraction of the categories mentioned by 46.9% of the interviewees about this problem. Mainly, they refer to the residual limb presenting pressure marks when it is pressed into the socket due to the external weight they must carry. They also indicate that they must distribute the weight they used to carry on a single journey (when they were not amputees) over multiple journeys. A situation that decreases their work efficiency and increases their fatigue when performing a repetitive task.

“On a trip like that, a healthy person can carry 6 or 7 *arrobas (1 arroba = 11.502 kg)*, but the way I am, I can make trips with only 2 or 2 and a half *arrobas*”. (S31, age58).

“Those storage sacks … you always must use a lot of strength to lift them. Before I was an amputee, they didn’t weigh much, but now I only lift what the prosthesis allows me to lift. I have lifted things that I have to lower to the ground and break them into pieces because I feel that the prosthesis can’t hold them, and they will fall off”. (S49, age80).

Others opt to ask someone else for help to perform these activities, which can also increase the costs of their farming activities.

“The storage sacks are loaded onto the truck after the harvest. However, I can’t carry them because I can’t hold the full weight of 50kg. The sacks are loaded by other guys and sometimes I must pay them”. (S11, age54).

“Mm… the spraying, I don’t do it myself, I must find someone else because the pump is heavy…”. (S32, age46).

In addition, they report balance problems when carrying external weight. They must lean more on their sound leg, thus overstressing their joints. One option to lighten the load is to use external elements such as a cane, but this limits them to using only one of their arms to support the weight.

“And sometimes I use the cane to carry it, so I don’t put too much weight on my leg (aiming at the prosthesis)”. (S3, age48).

The main agricultural activities in which this problem manifests itself are related to harvesting, watering plants, collecting firewood, spraying, distributing the harvest for sale, and transporting animal feed.

“When spraying…, I do 4 pumping, I do the irrigations and then I must rest because I’m not able to stand all day doing just that. The same is true for harvesting, watering plants and transporting animal feed.”. (S40, age32)

#### 3.2.6. Kneeling down

Although transtibial amputees retain the knee joint, 46.9% of amputees report problems with kneeling. One of the main causes of this problem is that the back of the knee is pressed by the socket when the knee is flexed.

“In the back part (behind the knee) the prosthesis wall was very high, you know what I mean? So, when I bend my leg, the pressure causes the stump to come out”. (S48, age24).

Some amputees choose to extend the knee of the prosthetic leg and flex only the sound knee (Figure 5(a)).

“I think that this (the amputated leg) does not allow me to kneel, I have almost never knelt with both (legs), I always kneel with one (the healthy limb) and stretch the other (the prosthetic leg)”. (S35, age29).

Respondents also show discomfort when kneeling for a long time, so they must opt to use supplementary elements such as knee braces or constantly change posture.

“To kneel? Well, I kneel, and I pray for 10 minutes, and after 10 minutes I must stand up because it starts to leave marks on my leg. It starts to hurt down here (behind the knee)” (S34, age40).

**Figure 9.**
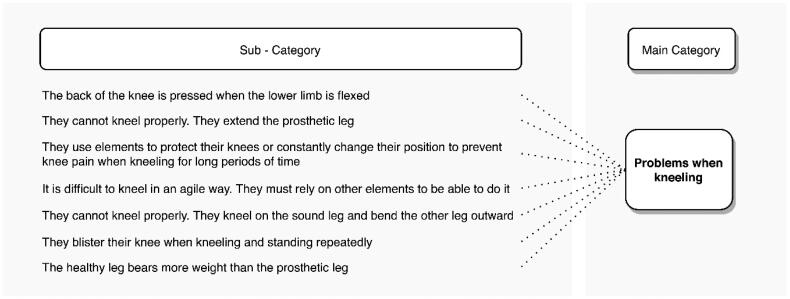
Problems reported by transtibial amputees while kneeling.

As in other postures, they bear more weight on the sound limb. These problems are mainly evidenced in agricultural activities such as planting, weeding, and fertilizing different crops, being coffee crops the most mentioned. The categories found in the abstraction process of the problems associated with kneeling are shown in Figure 9.

“Mm, when I fertilise the coffee plants, I get blisters on the back of my knee because I must stand up and kneel, stand up and kneel again, because that’s how this task is done”. (S1, age28).

“I must kneel and when I’m kneeling, I must change position because I get a sore on my knee joint from the prosthesis pressing against it. So sometimes I bend down and sometimes I kneel to be able to endure the discomfort”. (S1, age28).

#### 3.2.7. Standing for a long time

**Figure 10.**
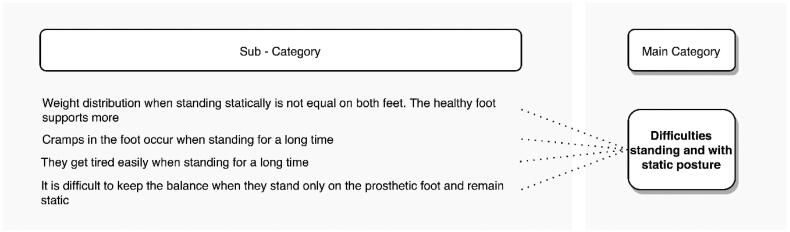
Problems reported by transtibial amputees when standing for a long time.

Prolonged standing is another posture in which transtibial amputees report problems. Figure 10 shows the subcategories associated with this posture, mentioned by 38.8% of the interviewees. They indicate that their feet cramp and they get tired easily. One of the compensations they make to alleviate the discomfort is to distribute their body weight unequally on both feet, with the healthy limb always bearing more weight. This causes pain in the sound leg and in the back.

“When I stand for a long time, it doesn’t affect me, but I don’t lean much on the prosthetic leg, but more on the sound leg…”. (S3, age48).

“I have pain in my back and pain in the sound limb because I always lean more on the sound limb”. (S19, age50).

“Normally I try not to put so much weight on the right side where I have the good foot, but I try to keep the weight balanced, because when I put the weight on the right side (non-amputated leg), my back hurts a lot. So now I try to keep the weight centred”. (S20, age30).

When grouping the participant quotes from the interviews by agricultural activity, there was no activity directly mentioned in this posture.

#### 3.2.8. Sitting down

**Figure 11.**
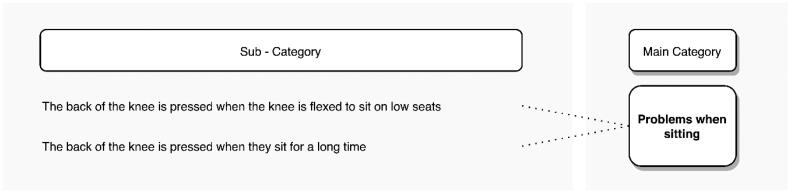
Problems reported by transtibial amputees when sitting.

Respondents also reported problems with sitting. Figure 11 shows the 2 subcategories associated with this posture. 18.4% of the interviewees indicated problems when sitting. The main cause being discomfort in the back of the knee when sitting on low seats or for a long time.

“When you sit down. I mean, when I sit on a low seat, I must stretch it (the prosthetic leg), because the part (the socket), where the stump goes, is uncomfortable (aiming at the back of the knee). It (the socket) does not expand so there is no room for the stump to fit properly”. (S28, age52).

Although this posture can be transversal to several agricultural activities, discomfort was mentioned mainly during milking. Some amputees choose to sit with the prosthetic leg straight to avoid discomfort in the posterior knee area.

“When milking you must sit very low. The cow will never be tall enough for me to sit high, … So, I cannot sit like a normal person would. I would not be able to sit well like that (sitting). Then I would have to have it stretched (the prosthetic leg) …, I stretch it and then I sit on the low seat” (S35, age29).

#### 3.2.9. Other postures in which transtibial amputees present problems

A minority of amputees mentioned problems performing activities such as running (8.2%), jumping (4.1%) or standing on tiptoes (2.1%). They mentioned that they cannot propel themselves well with the prosthetic leg when running due to the lack of articulation in the prosthetic foot. They cannot jump with the prosthesis because it is difficult for them to propel themselves.

“The only thing that bothers me is that I can’t push myself up or jump. The most difficult thing for me is jumping up and down.”. (S7, age52).

Sometimes they must climb trees during their farming activities and, instead of jumping, they rely on external elements to climb down. They also report that there is pressure behind the knee when they try to stand on tiptoes while wearing the prosthesis.

“Well… What I mentioned before … it’s hard when I must try to stand on my tiptoes or to force myself to stand upright. It bothers a little bit in the knee and behind the knee.” (S10, age56).

The main agricultural activities associated with these problems are caring for livestock, pruning trees, or reaching for objects that exceed the height of the person.

“With the prosthesis I have, it is very difficult for me to run. I can’t run behind an animal. I must rope the animal because I am not able to run. This probably happens because the foot is very stiff. The bottom of the foot is very stiff (aiming at the prosthesis)”. (S15, age36)).

## 4. Discussion

We were able to interview 49 transtibial amputees who work in the agricultural sector. Other studies such as the one by Waldera et al. [14] interviewed 40 farmers, of whom only 23 had lower limb amputations, where they considered transfemoral and transtibial amputations without discriminating the responses of the needs by type of amputation. This evidences that our work has shown the largest and most homogeneous sample to date of the transtibial amputees interviewed. The prevalence of men in the interviews (91.8%) is related to what was said by Brown [22], who indicated that 87% of the non-fatal amputations reported in 1999 involved men. Furthermore, according to the Society of Colombian Farmers (SAC), agricultural Colombian workers are predominantly male (84%) [23].

In the interviews, the agricultural tasks carried out by the farmers and the associated problems were inquired. However, it was not possible to classify the problems by type of specific agricultural activities. This limitation is since in the Colombian context, farmers tend to alternate the tasks of their working day between agricultural and livestock activities.

In the categorization of the information, mobility, and postural limitations common to various agricultural activities were found. The most referred is associated with walking (85.7% of the interviewees mentioned it), this is because walking is one of the main locomotor activities of the human being and the problems presented in this activity increase the degree of disability of the amputee [24]. In addition, this statement is also in agreement with what was stated by Willkomm et al. [25], who reported falls and injuries when walking on uneven, sloping terrain or icy surfaces. Waldera et al. [14] indicate that it is difficult for amputees to walk on uneven ground because they cannot accommodate their prosthetic foot well. In our research we were able to identify the most problematic surfaces, which are stony, swampy, tall grass and sloping terrain, the latter being very common in Colombian territory, especially in the Andean zone [26].

Regarding the functional assessment of unilateral transtibial amputees, Baron reported that most amputees are at a K2 or K3 level of mobility, indicating that, in general, unilateral transtibial amputees are autonomous in their mobility [27]. However, they face challenges when climbing up and down stairs or walking on slippery surfaces. Also, Baron indicated that these patients find it easier to walk in enclosed spaces and on sidewalks, as well as to sit down and stand up from elevated seating [27]. Our findings associated with open-field mobility challenges create a new need to assess the workspaces of farmers with transtibial amputation. It is important to carry out a biomechanical study conducted within the appropriate systems contexts including not only the individual/work demands but also organisation and psychosocial environments, to improve the design requirements of transtibial prostheses for agricultural amputees.

The second most mentioned group of problems is associated with discomfort in the skin of the stump due to the postures of agricultural work, 71.4% of the amputees associated it with causes external to the prosthesis, such as sweating due to the weather and the agricultural activities they carry out, the swampy terrain, and the mismatch in the socket. These problems can be a consequence of the design and materials with which the prostheses are made. Willkomm et al. [25] mention the need for a socket with adequate ventilation to work in this type of activity. This is a requirement that can reduce the frequency of problems associated with the skin of the stump, but it must be considered that ventilation must not sacrifice the resistance, durability, and cost of the prosthesis, which are main characteristics for this type of amputees as mentioned by Waldera [14].

There were also problems in postures such as squatting, kneeling, standing for a long time, sitting, using means of transport and, to a lesser extent, running, jumping and stand on tip toes. When analysing the answers presented by the amputees in the interviews, it was possible to deduce that the frequency with which they perform each position depends on the demand for them in different agricultural tasks. There are more common activities that require the farmer to kneel or squat, such as planting, composting, and weeding of crops. That is the reason they present a higher percentage in the analysis. However, postures such as jumping, standing on tiptoes, and running are activities that can be associated with tasks that many of the amputees do not dare to perform for fear of injury or a fall. Consequently, they do not refer to problems associated with these postures, which suggests that the realization of them is not very essential in this field of work.

Most of the interviewees in this study had prostheses manufactured by the Mahavir Kmina Artificial Limb Centre corporation: conventional prosthesis (exoskeletal resin structure) with rigid ankle, which meet the conditions mentioned by other authors, such as mechanical resistance, cost/durability ratio, impermeability, and resistance to corrosion by agents inherent to the work environment [14,25]. However, they still present many problems that are caused both by external agents and by functional problems of the prosthesis itself. The useful life of these devices is reduced in amputees in rural areas. According to professionals and amputees interviewed, a prosthesis that lasts 3 years in the city can last 1 year or less when used in agricultural work. Amputees who have used both modular and conventional prostheses indicate that the latter are more appropriate for field work. However, those presents kinematics problems that need to be thoroughly evaluated.

## 5. Conclusions

In conclusion, the prostheses worn by transtibial amputee farmers are not suitable for working on sloping and uneven terrain, nor for performing postures such as kneeling or squatting. These postures are very common in agricultural and livestock tasks in countries with mountainous areas such as Latin American countries.

The recognition of mobility and postural limitations reported by transtibial amputees who perform agricultural tasks, may help to improve the design of prostheses so that they meet the needs of this population and decrease secondary injuries associated with prosthetic use. This information is useful to identify compensatory postures that facilitate prosthetic adaptation and rehabilitation for amputees. However, it is important to carry out a biomechanical study conducted within the appropriate systems contexts including not only individual and work demands but also organisation and psychosocial environments, to improve the design requirements of transtibial prostheses for agricultural amputees.

Further research is needed on the influence of prosthetic device experience on amputee mobility and postural limitations in various agricultural activities. This will provide better information for redesigning such devices or improving the working conditions of these people.

## Data Availability

The data that support the findings of this study are openly available in 4TU.ResearchData at http://doi.org/10.4121/54fed3ba-8cf0-404b-9e83-dfd4a4eb4d34.

## Notes

1 The verbal and written consents were approved by the ethics committee for the telephone interviews, due to the health contingency at the time of the interviews (2020–2022). These consents were audio-recorded at the beginning of each interview, indicating the approval of each interviewee to participate in the study.

2 The data of this research can be requested to the corresponding author.

## Geolocation information

This research was carried out in Medellín and its metropolitan area. Information was collected throughout the Colombian territory, mainly in Antioquia.
